# Untargeted metabolomic profiling in acute ischemic stroke patients with cerebral microbleeds

**DOI:** 10.3389/fneur.2025.1656974

**Published:** 2025-09-12

**Authors:** Lintao Zhou, Huifang Sun, Xiang Li, Qing Han

**Affiliations:** ^1^Department of Neurology, The First Affiliated Hospital of Ningbo University, Ningbo, China; ^2^Department of Internal Medicine, Yin Zhou NO.3 Hospital, Ningbo, China

**Keywords:** stroke, cerebral microbleeds, metabolomics, cerebral small vessel disease, mechanisms

## Abstract

**Background:**

Acute ischemic stroke (AIS) is a common cerebrovascular condition. Cerebral microbleeds (CMBs) are frequently observed in AIS patients and are closely associated with poor prognosis and potential therapeutic implications. Understanding the distinct metabolic profiles in AIS patients with CMBs is critical for uncovering the underlying pathophysiological mechanisms and identifying novel biomarkers.

**Methods:**

An untargeted metabolomics approach using liquid chromatography–mass spectrometry (LC–MS) was employed to compare the metabolic profiles of 30 AIS patients with CMBs (CMB group) and 30 AIS patients without CMBs (the Non CMB group, abbreviated as NCMB group). Raw MS data were processed using MS-DIAL and metabolites were identified by comparison with public and in-house databases. Both univariate and multivariate analyses (PCA, OPLS-DA) were used to identify differential metabolites, followed by KEGG pathway enrichment analysis.

**Results:**

The LC–MS platform demonstrated robust stability and high data quality. Multivariate statistical modeling successfully distinguished between the two groups, revealing distinct metabolic phenotypes. A total of 156 significantly altered metabolites were identified, including 103 upregulated and 53 downregulated metabolites. Pathway analysis revealed significant perturbations in lipid metabolism, amino acid metabolism, and energy metabolism.

**Conclusion:**

This study identified unique metabolic signatures in AIS patients with CMBs. The metabolites such as N-ethylglycine, aspartyl-glutamate, and oleamide were significantly elevated, while metabolites like PC (16:0/18:1) and PC (18:0/20:4) were significantly reduced, and other metabolites implicated disruptions in energy and lipid metabolism. These findings suggest potential biomarker candidates for diagnosis, prognosis, and therapeutic intervention in this high-risk population.

## 1 Introduction

Acute ischemic stroke (AIS) is a leading cause of disability and death worldwide. Among AIS patients, cerebral microbleeds (CMBs) are frequently detected and have been associated with adverse clinical outcomes. CMBs are defined as small hypointense lesions visible on susceptibility-weighted imaging (SWI) (1), representing microvascular hemorrhages due to structural damage to cerebral small vessels (2).

Although the exact mechanisms underlying CMB formation remain unclear, multiple pathophysiological processes have been proposed, including inflammation, oxidative stress, and blood–brain barrier (BBB) disruption (3). White matter hyperintensities (WMHs), another marker of cerebral small vessel disease (CSVD) (4), are also linked to similar pathological mechanisms (5). While some studies have reported no significant association between CMBs and microglial activation or BBB permeability, animal and neuropathological studies suggest a potential role of neuroinflammation (1).

Metabolomics offers a comprehensive and dynamic overview of low-molecular-weight metabolites within biological systems. Unlike genomics or proteomics, metabolomics reflects downstream biochemical activity, providing insights into real-time physiological states (6). This approach is particularly valuable for investigating complex diseases like AIS and CSVD (7). Metabolomics has been emphasized by Lasica et al. as a powerful tool for monitoring secondary brain injury and complex pathological processes following cerebrovascular events, such as aneurysmal subarachnoid hemorrhage, enabling the elucidation of dynamic disease progression (8). More significantly, this approach demonstrates considerable potential in identifying subtype-specific biomarkers for various cerebrovascular diseases. In a large-scale prospective study of Chinese adults, distinct plasma metabolite profiles were found to be strongly associated with incident ischemic stroke and its subtypes (9). These findings robustly suggest that different cerebrovascular pathological substrates (e.g., atherosclerosis or small vessel disease) may possess unique metabolic fingerprints.

Despite the clinical significance of CMBs in AIS, the specific metabolic alterations associated with CMBs remain poorly characterized. This study aimed to apply an untargeted LC–MS-based metabolomics strategy to identify metabolic differences between AIS patients with and without CMBs. To our knowledge, this represents the first study in recent years to employ untargeted metabolomics for comparing AIS patients with and without CMBs, revealing distinct metabolic disturbance patterns specific to CMBs. The study aimed to identify distinct metabolic signatures and dysregulated pathways that could serve as novel targets for CMB diagnosis, early risk stratification, and personalized therapeutic strategies.

## 2 Materials and methods

### 2.1 Study design and patient cohort

This cross-sectional case–control study recruited AIS patients from the Department of Neurology, First Affiliated Hospital of Ningbo University, Ningbo city, Zhejiang province, China, between January and September 2024. A total of 60 AIS patients were enrolled and classified into two groups: 30 with CMBs (CMB group) and 30 without CMBs (NCMB group).

Inclusion criteria for the CMB group were: admission within 7 days of onset, age ≥18 years, diagnosis of AIS confirmed by diffusion-weighted imaging (DWI), and presence of ≥1 CMB on SWI. The inclusion criteria for the non-CMB (NCMB) group were identical to those of the CMB group, with the exception of the absence of microbleeds on susceptibility-weighted imaging (SWI). Both groups shared the same exclusion criteria. Exclusion criteria included autoimmune disorders, malignancy, severe infections, significant hepatic or renal dysfunction, or incomplete clinical/imaging data. The study protocol was approved by the ethics committee of the First Affiliated Hospital of Ningbo University(Approval NO.: the First Affiliated Hospital of Ningbo University LUNSHEN 2024 Research No. 066A; Approval date: June 28, 2024).

### 2.2 Clinical and imaging data collection

All patients underwent neuroimaging, including 1.5 T brain MRI and vascular imaging. The neuroimaging data were independently evaluated by two board-certified neurologists, each with over a decade of specialized experience in stroke neurology and specific expertise in interpreting susceptibility-weighted imaging (SWI) for cerebral microbleed (CMB) detection. Demographic and clinical data were collected, including age, sex, smoking and drinking history, vascular risk factors (hypertension, diabetes, dyslipidemia, coronary artery disease), NIH Stroke Scale (NIHSS) scores, and modified Rankin Scale (mRS) scores. Laboratory tests included blood counts, eGFR, total cholesterol (TC), triglycerides (TG), HDL-C, LDL-C, and HbA1c.

### 2.3 Sample preparation and untargeted metabolomics analysis

EDTA-containing blood samples of all patients were collected after fasting overnight and then centrifuged within 30 min after blood draw at 3,000 rpm for 15 min at 4°C. These serum samples were stored immediately at −80°C until analyses and avoid repeated freezing and thawing during storage.

100 μl serum was thoroughly mixed with 400 μl of cold methanol acetonitrile (v/v, 1:1) via vortexing. And then the mixture were processed with sonication for 1 h in ice baths. The mixture was then incubated at −20°C for 1 h, and centrifuged at 4°C for 20 min with a speed of 14, 000 g. The supernatants were then harvested and dried under vacuum LC–MS analysis.

Metabolomics profiling was analyzed using a UPLC-ESI-Q-Orbitrap-MS system (UHPLC, Shimadzu Nexera X2 LC-30 AD, Shimadzu, Japan) coupled with Q-Exactive Plus (Thermo Scientific, San Jose, USA).

For liquid chromatography (LC) separation, samples were analyzed using a ACQUITY UPLC® HSS T3 column (2.1 × 100 mm, 1.8 μm; Waters, Milford, MA, USA). The flow rate was 0.3 ml/min and the mobile phase contained: A: 0.1% FA in water and B: 100% acetonitrile (ACN). The gradient was 0% buffer B for 2 min and was linearly increased to 48% in 4 min, and then up to 100% in4 min and maintained for 2 min, and then decreased to 0% buffer B in 0.1 min, with 3 min re-equilibration period employed.

The electrospray ionization (ESI) with positive-mode and negative mode were applied for MS data acquisition separately. The HESI source conditions were set as follows: Spray Voltage:3.8kv (positive) and 3.2kv (negative); Capillary Temperature:320°C; Sheath Gas (nitrogen) flow: 30 arb (arbitrary units); Aux Gas flow: 5 arb; Probe Heater Temp: 350°C; S-Lens RF Level:50. The instrument was set to acquire over the m/z range 70–1,050 Da for full MS. The full MS scans were acquired at a resolution of 70,000 at m/z 200, and 17,500 at m/z 200 for MS/MS scan. The maximum injection time was set to for 100 ms for MS and 50 ms for MS/MS. The isolation window for MS2 was set to 2 m/z and the normalized collision energy (stepped) was set as 20, 30 and 40 for fragmentation.

### 2.4 Data processing and metabolite identification

Raw MS data were processed using MS-DIAL software for peak alignment, retention time correction, and peak area extraction. To monitor analytical variability, quality control (QC) samples (pooled from aliquots of all study samples) were injected every 7–8 experimental samples and processed identically. No internal standards were employed. Signal drift was addressed during data preprocessing via total peak area normalization (performed separately for positive and negative ion modes) and retention time correction in MS-DIAL.

Metabolites were identified by matching accurate mass (mass tolerance <10 ppm) and MS/MS spectra (mass tolerance <0.02 Da) against public databases (HMDB, MassBank, GNPS) and an in-house library (BP-DB). For downstream analysis, ion peaks with >50% missing values within any group were excluded. Integrated positive and negative ion data were subjected to Unit Variance Scaling (UV) preprocessing in Python prior to statistical modeling.

### 2.5 KEGG enrichment analysis

To identify the perturbed biological pathways, the differential metabolite data were performed KEGG pathway analysis using KEGG database.^1^ KEGG enrichment analyses were carried out with the Fisher’s exact test, and FDR correction for multiple testing was performed. Enriched KEGG pathways were nominally statistically significant at the *p* < 0.05 level.

### 2.6 Statistical analysis

Statistical analysis was conducted using R software (v4.4.2). Continuous variables were expressed as medians (IQR) and compared using the Mann–Whitney U test. Categorical variables were presented as counts (percentages) and analyzed using the chi-square test. Pearson correlation was used for assessing relationships between continuous variables.

Differential metabolites were identified using both univariate and multivariate approaches. Selection criteria included OPLS-DA variable importance in projection (VIP) > 1 and *p*-value <0.05, or fold change (FC) ≥ 1.5 or ≤1/1.5 with *p* < 0.05. OPLS-DA models were validated via 200-time permutation tests. All plots were generated using R.

## 3 Results

### 3.1 Demographics and clinical characteristics

The baseline characteristics of the CMB and NCMB groups are summarized in Table 1. No statistically significant differences were observed between the groups in terms of age, sex, body mass index (BMI), hematological parameters, estimated glomerular filtration rate (eGFR), lipid profiles, HbA1c, admission NIHSS scores, or mRS scores. However, hypertension, diabetes, coronary heart disease, smoking, and alcohol consumption were significantly more prevalent in the NCMB group (*p* < 0.05; Table 1).

**Table 1:** Baseline characteristics of acute ischemic stroke patients with and without cerebral microbleeds.

Variable	MCB (*N* = 30)	NCMB (*N* = 30)	Overall (*N* = 60)	*p*-value
Age, years	69.5 (64.0, 77.0)	71.0 (66.0, 80.0)	70.0 (64.0, 77.0)	0.999
Sex	13 (43.3%)	16 (53.3%)	29 (48.3%)	0.354
BMI	24.5 (21.5, 27.7)	22.9 (20.4, 24.7)	23.9 (21.1, 27.2)	0.179
NIHSS	2.0 (1.0, 3.0)	1.5 (0.0, 3.0)	2.0 (1.0, 3.0)	0.203
mRS	1.0 (1.0, 2.0)	1.0 (1.0, 1.0)	1.0 (1.0, 2.0)	0.244
Hypertension	23 (76.7%)	29 (96.7%)	52 (86.7%)	0.004
Diabetes	8 (26.7%)	19 (63.3%)	27 (45.0%)	0.004
CHD	2 (6.7%)	7 (23.3%)	9 (15.0%)	0.043
Smoking	6 (20.0%)	19 (63.3%)	25 (41.7%)	0.004
Drinking	7 (23.3%)	19 (63.3%)	26 (43.3%)	0.004
WBC	6.1 (5.3, 7.0)	6.4 (5.5, 7.4)	6.2 (5.4, 7.2)	0.443
RBC	4.3 (4.1, 4.7)	4.4 (4.1, 4.8)	4.3 (4.1, 4.7)	0.771
eGFR	81.7 (67.0, 99.0)	82.0 (66.5, 96.0)	82.0 (67.0, 96.0)	0.963
TG	1.1 (0.7, 1.5)	1.3 (0.9, 1.6)	1.2 (0.8, 1.6)	0.354
TC	4.2 (3.4, 5.2)	4.3 (3.7, 5.5)	4.3 (3.5, 5.3)	0.655
LDL	2.7 (2.1, 3.5)	2.8 (2.1, 3.5)	2.7 (2.1, 3.5)	0.999
HDL	1.0 (0.9, 1.2)	1.1 (1.0, 1.2)	1.1 (0.9, 1.2)	0.179
HbA1C	6.1 (5.6, 7.2)	6.2 (5.5, 7.3)	6.1 (5.6, 7.2)	0.771

### 3.2 Quality control and system stability

Quality control assessments demonstrated excellent system stability and analytical reproducibility. QC samples prepared from pooled aliquots consistently validated retention time alignment and peak intensity normalization. These measures ensured that observed differences in metabolomic profiles were attributable to biological variation rather than technical artifacts.

### 3.3 Multivariate analysis of metabolic profiles

**Figure 1:**
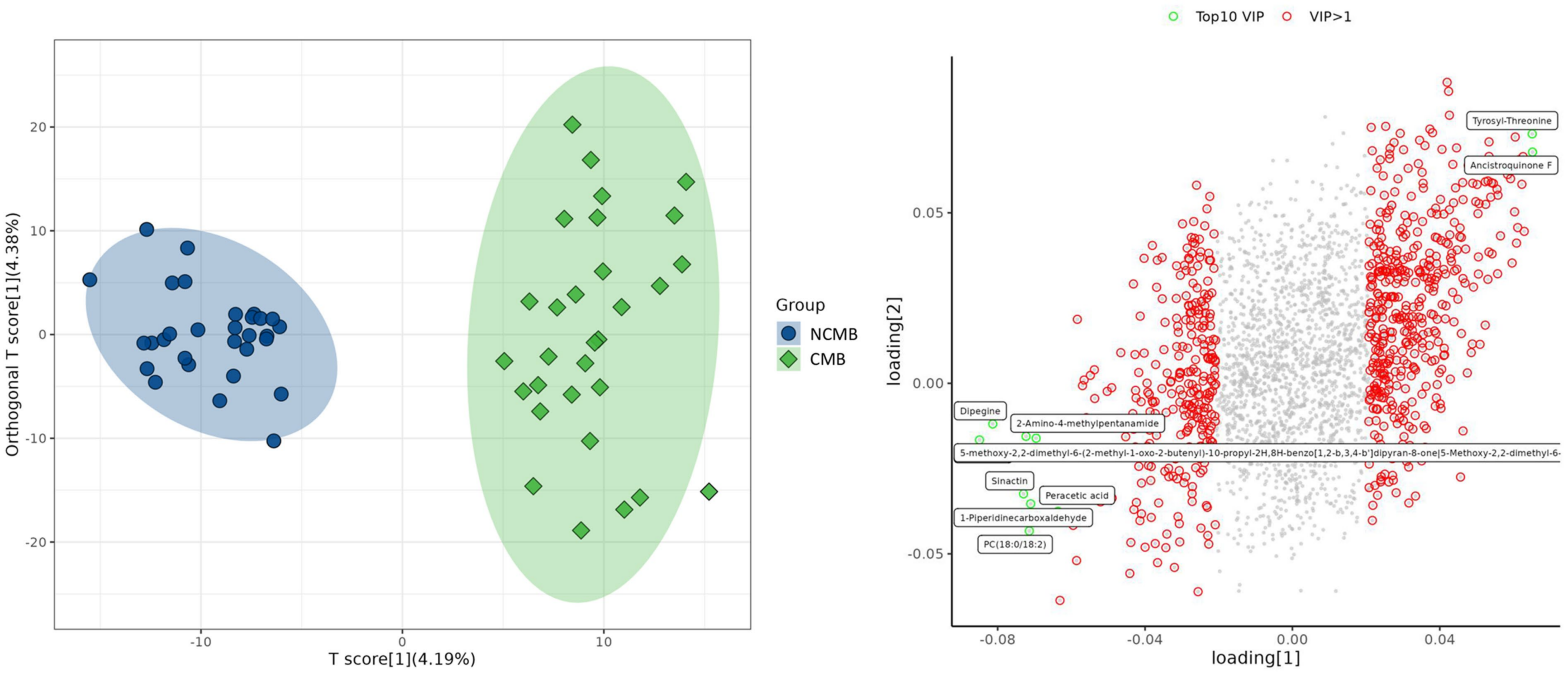
OPLS-DA score plot and loading plot of acute ischemic stroke (AIS) patients with and without cerebral microbleeds (CMB).

Orthogonal partial least squares discriminant analysis (OPLS-DA) indicated some degree of separation between the metabolic profiles of the CMB and NCMB groups. The model yielded statistics (R^2^X = 0.0858, R^2^Y = 0.934, Q^2^ = 0.573), suggesting that only a small proportion of the total variance was captured. Therefore, while the analysis provides exploratory insights and facilitated the identification of key metabolites contributing to group separation, the findings should be interpreted with caution (Figure 1).

### 3.4 Identification of differential metabolites

**Figure 2:**
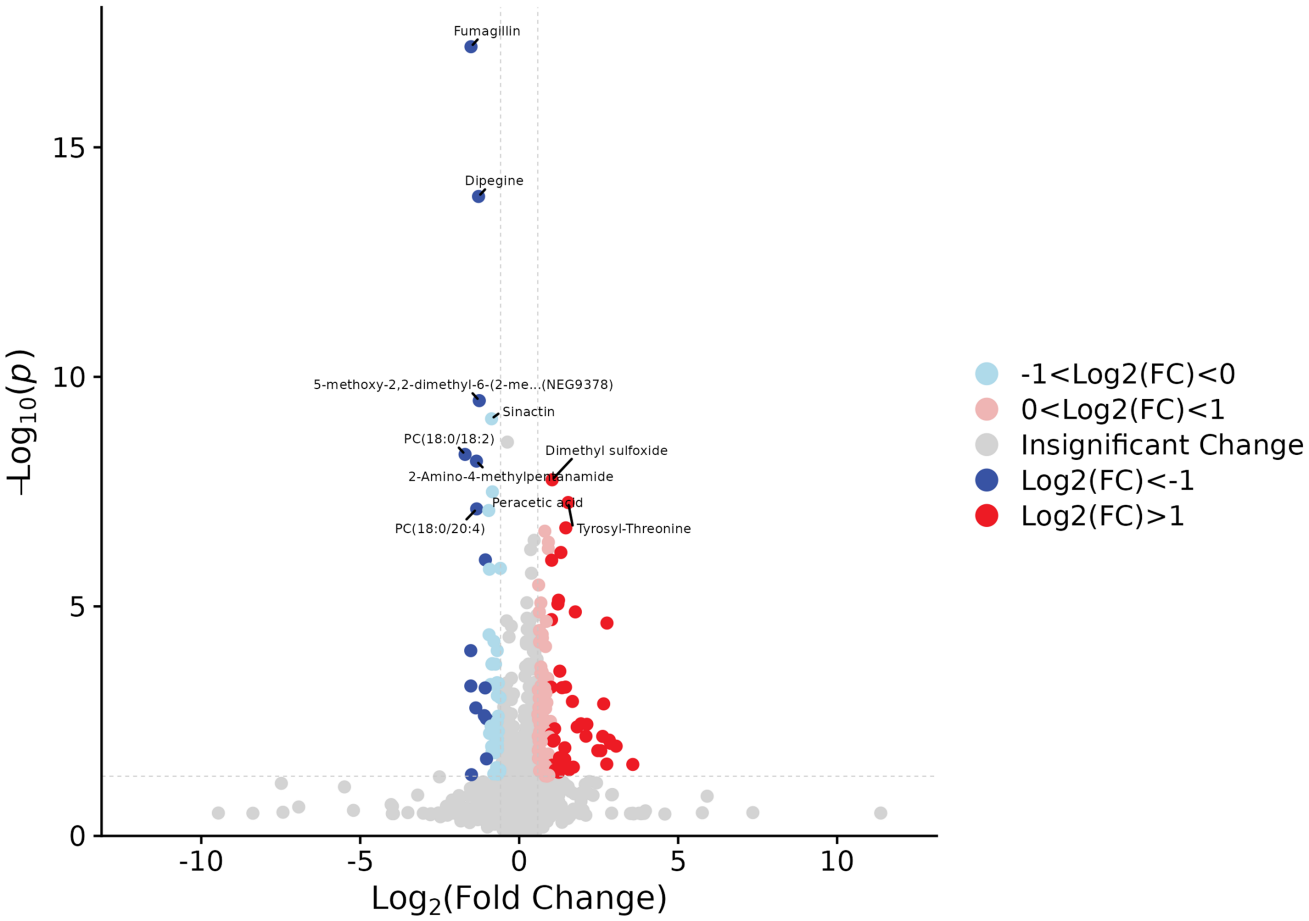
Differential metabolites identified by univariate analysis in acute ischemic stroke patients with and without cerebral microbleeds.

**Figure 3:**
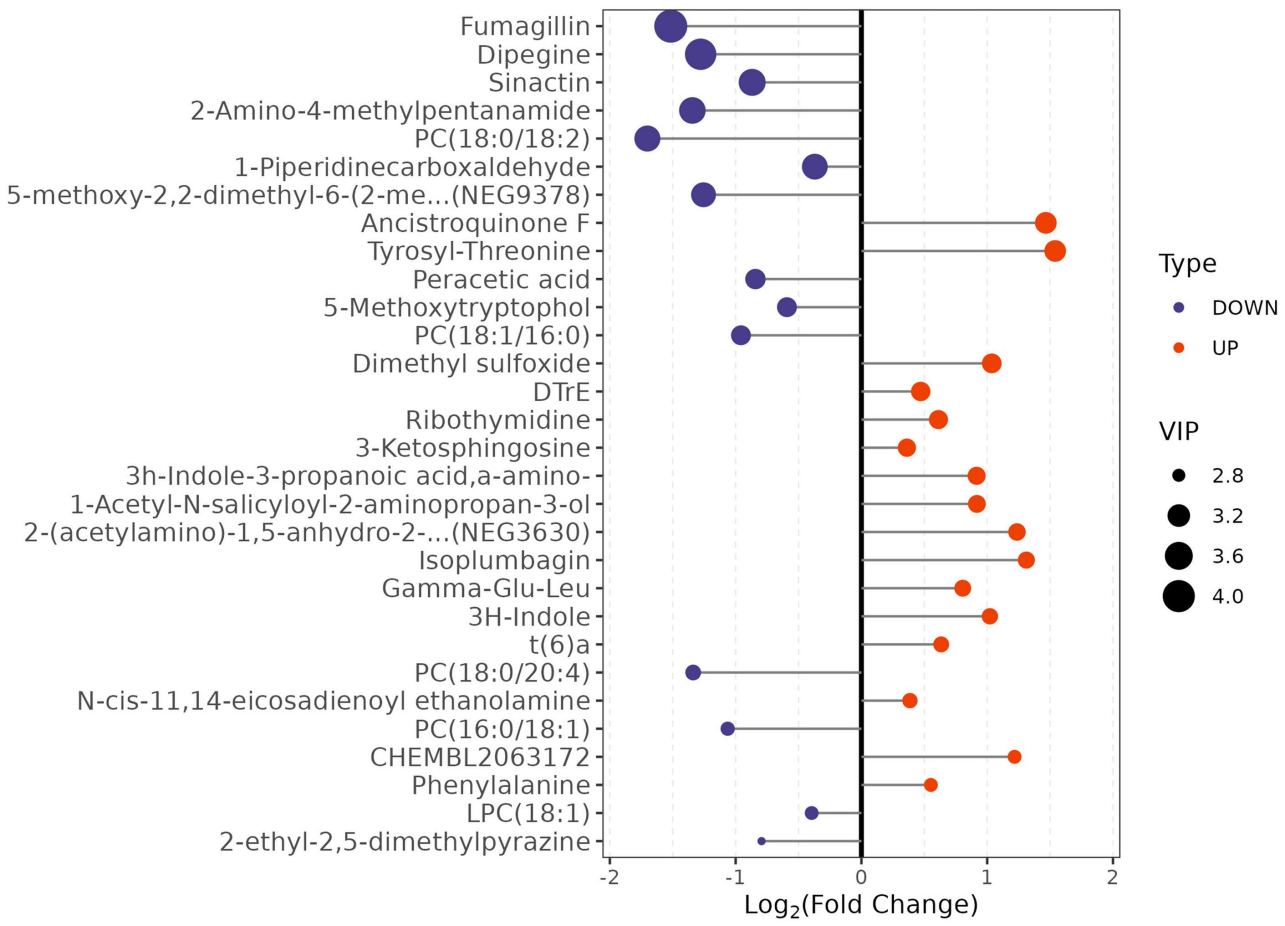
Top 30 metabolites with significant fold changes and VIP scores in AIS patients with vs. without cerebral microbleeds.

**Figure 4:**
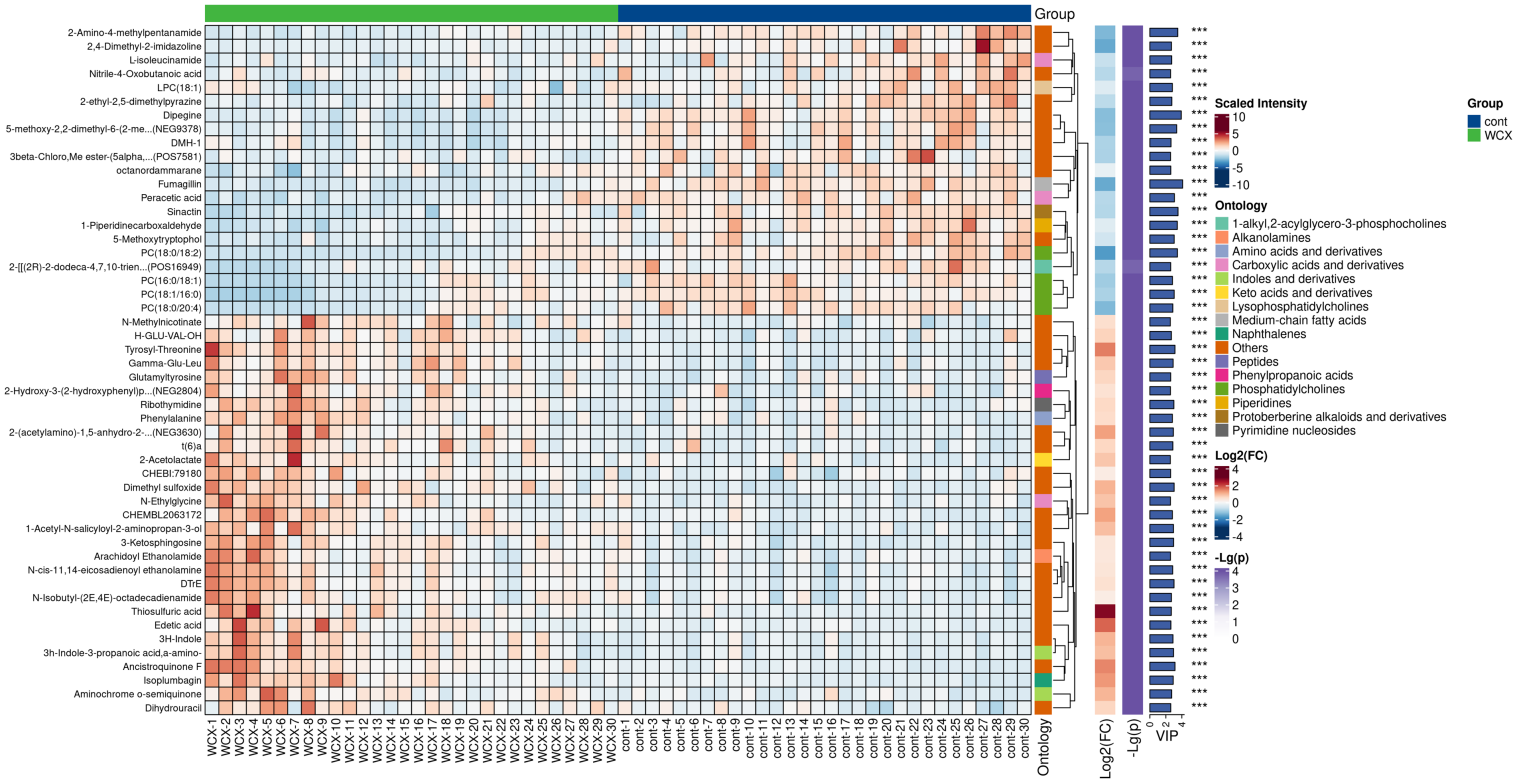
Hierarchical clustering analysis of differential metabolites in AIS patients with and without cerebral microbleeds.

**Figure 5:**
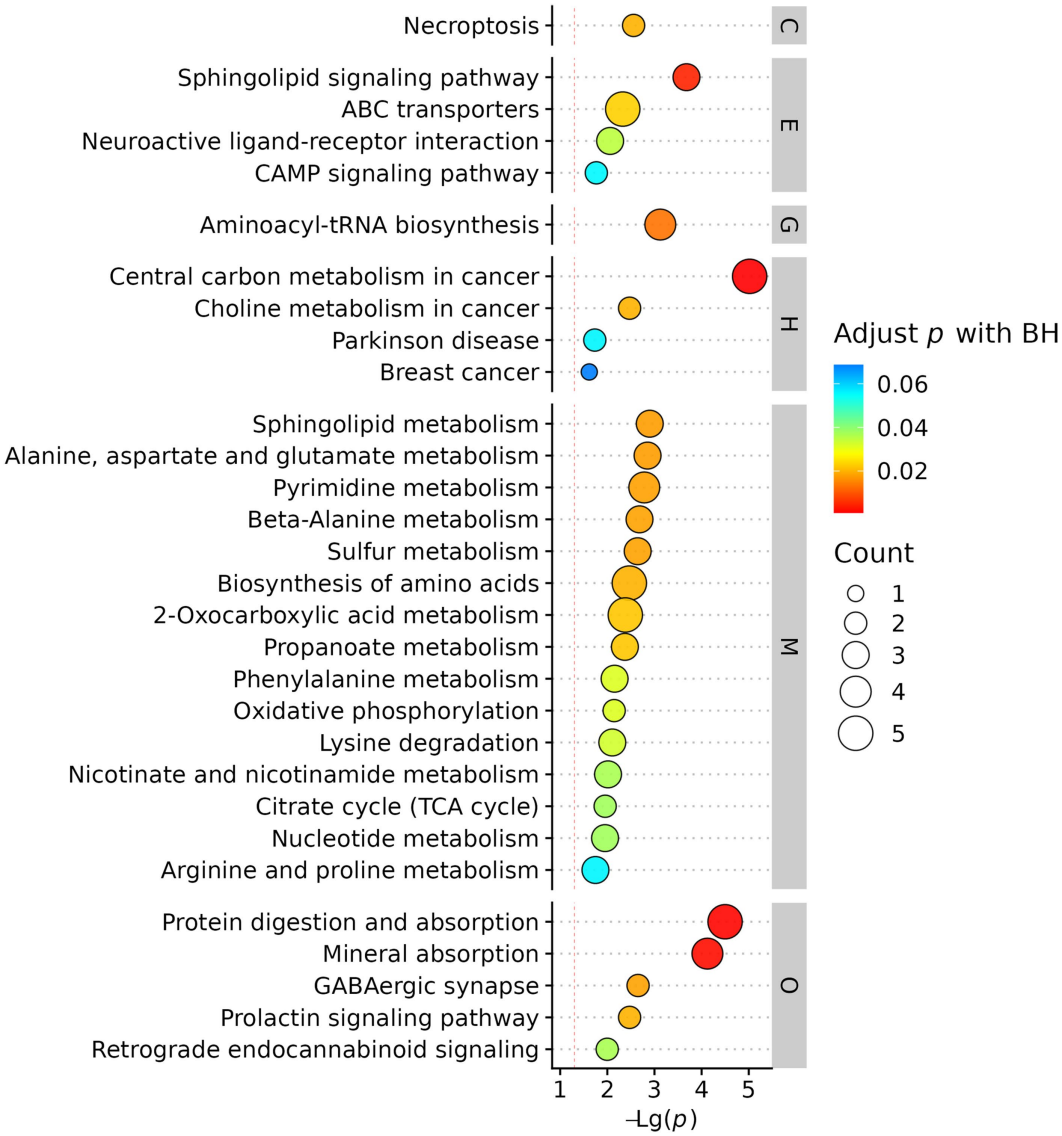
Enriched metabolic pathways in AIS patients with vs without cerebral microbleeds.

Based on predefined statistical thresholds (VIP > 1 and *p* < 0.05, or FC ≥ 1.5 or ≤1/1.5 and p < 0.05), a total of 156 differential metabolites were identified—103 upregulated and 53 downregulated in the CMB group. Notable upregulated metabolites included oleamide, methionine, dimethyl sulfoxide (DMSO), succinate, N-ethylglycine, tyrosine, γ-glutamylmethionine, O-arachidonoylethanolamine, LPE (18:0/0:0), leucine, and aspartylglutamate. These spanned various chemical classes, notably fatty acyls, amino acids, and glycerophospholipids. Downregulated compounds included N-(carboxymethyl)-N-(2-((carboxymethyl)amino)ethyl)glycine, PC (16:0/18:1) and PC (18:0/20:4), erythronic lactone, 2-cyanoacetamide, and GW 9662 (Figures 2–5; Supplementary Table S1).

### 3.5 Enriched metabolic pathways and functional analysis

**Figure 6:**
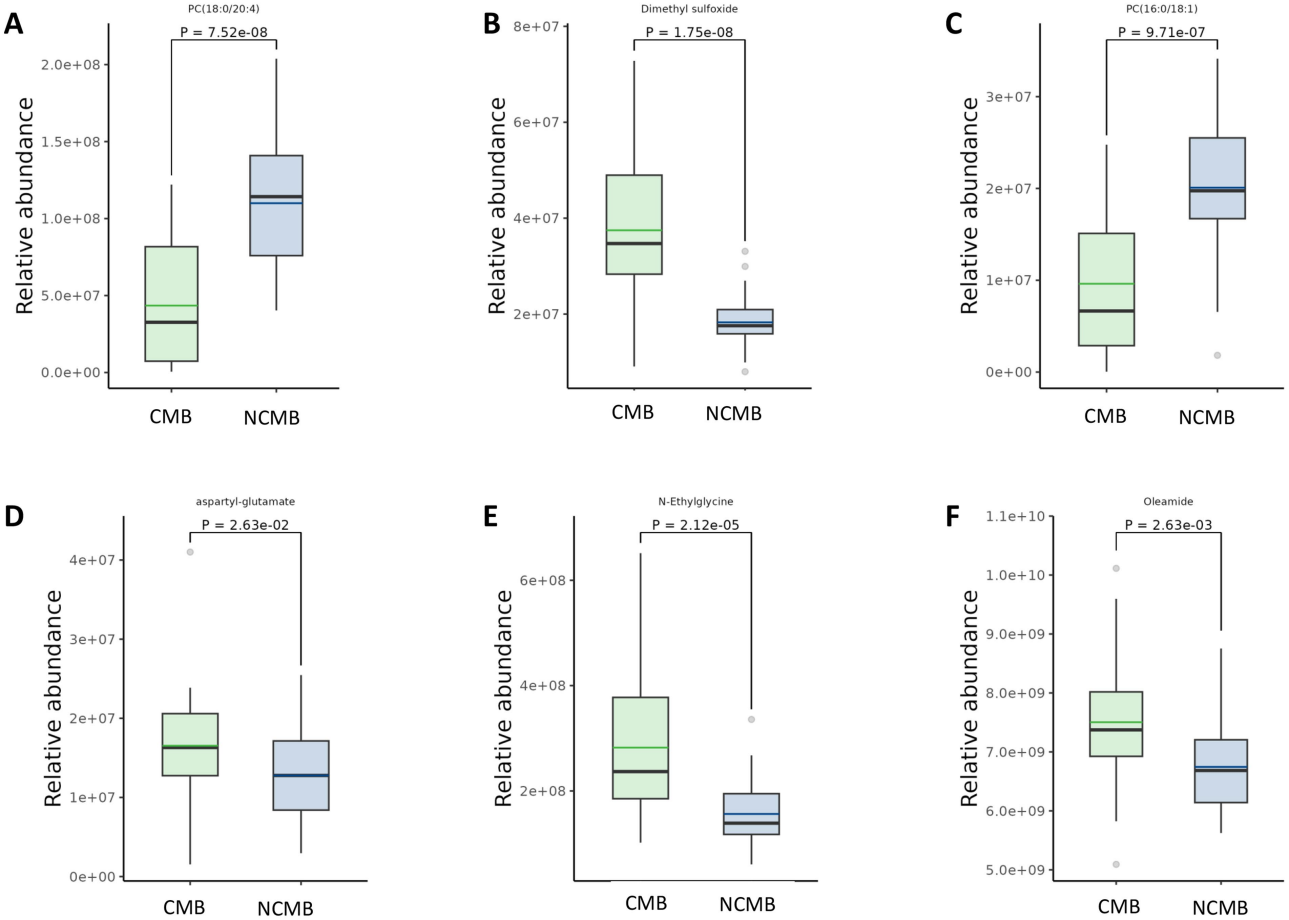
Comparison of differential metabolites in AIS patients with versus without cerebral microbleeds. Panels **(A,C)** depict metabolites that are significantly decreased in the CMB group relative to the Non-CMB group. Panels **(B,D–F)** depict metabolites that are significantly increased in the CMB group relative to the Non-CMB group.

KEGG pathway enrichment analysis mapped differential metabolites to biologically relevant pathways. The most significantly enriched pathways were associated with energy metabolism, oxidative stress, and inflammatory responses, indicating systemic metabolic disturbances in AIS patients with CMBs. (Figure 6).

## 4 Discussion

Distinct metabolic profiles of acute ischemic stroke (AIS) patients with cerebral microbleeds (CMB) were successfully identified through untargeted metabolomics analysis. Significant alterations in metabolic patterns were observed in CMB patients compared to non-CMB counterparts, involving multiple metabolite classes and underlying biological pathways.

The predominantly upregulated metabolites included amino acids (methionine, leucine, tyrosine) and organic acids (succinate, N-ethylglycine, aspartylglutamate), which are closely associated with energy metabolism pathways. Differential regulation was observed in the tricarboxylic acid (TCA) cycle, alanine-aspartate–glutamate metabolism, and pyruvate metabolism pathways, predominantly affecting energy homeostasis. The persistent upregulation or downregulation of energy-producing metabolites (e.g., TCA cycle intermediates, pyruvate, specific amino acids) suggests aberrant energy metabolism or mitochondrial dysfunction in CMB patients. These findings align with established mechanisms of neuronal injury in stroke, particularly cellular energy failure (10). The presence of specific organic acids may indicate metabolic blockades or shunting. Impaired energy metabolism likely exacerbates neuronal damage and impedes recovery in CMB patients, contributing to poorer clinical outcomes.

Numerous differentially expressed metabolites belonged to lipid categories, particularly fatty acyls and glycerophospholipids [e.g., upregulated oleamide and O-arachidonoylethanolamine; downregulated PC (O-16:0/20:5)]. Cerebral hemorrhage has been strongly associated with oxidative stress and elevated lipid peroxidation products (5). The substantial alterations in fatty acyls and glycerophospholipids strongly suggest disrupted lipid metabolism, implicating lipid peroxidation as a key pathological process in CMB.

Notably, a significant increase in a feature annotated as dimethyl sulfoxide (DMSO) was observed in the CMB group. DMSO is known to exhibit antioxidant properties and modulate cerebral metabolism, showing neuroprotective effects in experimental traumatic brain injury models (11). In humans, endogenous DMSO production remains controversial, with most evidence supporting the endogenous presence of its oxidized form, dimethyl sulfone (DMSO₂), derived from methanethiol metabolism. Although our sample preparation protocol did not involve the use of DMSO and procedural blanks did not show detectable peaks, the possibility of trace exogenous contamination or misannotation cannot be excluded. Therefore, this finding should be interpreted with caution. Ideally, future targeted LC–MS studies using authentic standards and isotope-labeled approaches are required to confirm the presence and biological relevance of DMSO in CMB patients.

Although some studies report no direct association between CMB and microglial activation/blood–brain barrier leakage, evidence suggests CMB can induce inflammatory responses (12). The identified lipid metabolites—particularly glycerophospholipids like LPE (18:0/0:0) and PC (18:0/20:4)—are known mediators of inflammation (13). Furthermore, downregulated erythronolactone, a lactone compound implicated in neuroinflammation (14), was observed. These metabolomic findings suggest altered inflammatory mediators/pathways in CMB, potentially indicating an inflammatory component—either as a cause or consequence of microbleeds. Inflammation adversely affects blood–brain barrier integrity (15), and elevated plasma inflammatory markers have been documented in lacunar stroke patients (16, 17). Pathological examinations frequently reveal inflammatory cell infiltrates in perforating arterioles and perivascular tissues adjacent to lacunes. Therefore, further analysis of these differential lipid metabolites may yield novel insights or molecular targets for future investigation.

The findings of this study hold significant translational potential. The key differential metabolites identified—including N-ethylglycine, aspartylglutamate, and oleamide—provide promising candidates for developing novel biomarker panels for CMB risk stratification and prognostic evaluation. Future longitudinal studies should track the dynamic changes in these metabolites to assess their utility in monitoring disease progression or therapeutic response. Such investigations will represent a critical step toward translating these fundamental discoveries into clinical applications, potentially enabling precision medicine strategies for AIS patients with CMBs.

## 5 Limitations

This study has several limitations that should be acknowledged. First, the relatively small sample size may restrict the generalizability of the findings. Second, the inclusion window of 7 days after stroke onset may increase heterogeneity in metabolic profiles. Third, no independent validation cohort was included, which limits the external validity of our results. Fourth, the cross-sectional design precludes causal inference and prevents assessment of temporal changes in metabolite levels. Fifth, the reliance on LC–MS alone, while providing robust coverage of lipids and amino acids, may have missed other metabolite classes (e.g., volatile organic compounds); future investigations could benefit from multi-platform metabolomic approaches. Additionally, the low R^2^X value (0.0858) indicates that our model explains only a small proportion of the total metabolic variance, suggesting the need for expanded sample sizes in subsequent research. Notably, significant baseline differences in hypertension, diabetes, coronary artery disease, smoking, and alcohol consumption—all more prevalent in the NCMB group—may introduce confounding effects. Future studies should employ more rigorously matched cohorts to address this limitation.

## 6 Conclusion

This study identified unique metabolic signatures in AIS patients with CMBs. The metabolites such as N-ethylglycine, aspartyl-glutamate, and oleamide were elevated, while metabolites like PC (16:0/18:1) and PC (18:0/20:4) were reduced, and other metabolites implicated disruptions in energy and lipid metabolism. These findings suggest potential biomarker candidates for diagnosis, prognosis, and therapeutic intervention in this high-risk population.

## Data Availability

The original contributions presented in the study are included in the article/Supplementary material, further inquiries can be directed to the corresponding author.

## Footnotes

^1^ http://www.kegg.jp
