# Supplementary material for: Untargeted metabolomic profiling in acute ischemic stroke patients with cerebral microbleeds
Source: Front Neurol. 2025 Sep 12;16:1656974. doi: 10.3389/fneur.2025.1656974 (PMC12506673; doi:10.3389/fneur.2025.1656974)
Supplement: Supplementary file 1 [file Table_1.DOCX]

Supplementary Table S1. Partial differential metabolites in AIS patients with CMBs group patients

| Classification | Metabolite name | FC | OPLSDA.VIP | P-value | Superclass | Class |
| --- | --- | --- | --- | --- | --- | --- |
| Elevated | Oleamide | 1.112 | 1.798 | 0.0026 | Lipids and lipid-like molecules | Fatty Acyls |
|  | Methionine | 1.194 | 1.450 | 0.0162 | Organic acids and derivatives | Carboxylic acids and derivatives |
|  | Dimethyl sulfoxide | 2.051 | 3.037 | <0.0001 | Organosulfur compounds | Sulfoxides |
|  | Succinic acid | 1.282 | 1.876 | 0.0051 | Organic acids and derivatives | Carboxylic acids and derivatives |
|  | N-Ethylglycine | 1.806 | 2.576 | <0.0001 | Organic acids and derivatives | Carboxylic acids and derivatives |
|  | Tyrosine | 1.420 | 2.137 | 0.0017 | Organic acids and derivatives | Carboxylic acids and derivatives |
|  | gamma-Glutamylmethionine | 1.414 | 1.535 | 0.0073 | Organic acids and derivatives | Carboxylic acids and derivatives |
|  | O-Arachidonoyl Ethanolamine | 1.426 | 2.101 | 0.0017 | Lipids and lipid-like molecules | Fatty Acyls |
|  | LPE(18:0/0:0) | 1.166 | 1.299 | 0.0442 | Lipids and lipid-like molecules | Glycerophospholipids |
| Reduced | PC(O-16:0/20:5) | 0.551 | 2.608 | 0.0002 | Lipids and lipid-like molecules | Glycerophospholipids |
|  | Erythronolactone | 0.745 | 1.670 | 0.0062 | Organoheterocyclic compounds | Lactones |
|  | 2-Cyanoacetamide | 0.839 | 1.284 | 0.030 | Organic acids and derivatives | Carboxylic acids and derivatives |
|  | PC(18:0/20:4) | 0.396 | 2.873 | <0.0001 | Lipids and lipid-like molecules | Glycerophospholipids |
|  | PC(16:0/18:1) | 0.478 | 2.828 | <0.0001 | Lipids and lipid-like molecules | Glycerophospholipids |
